# Cortical Activations in Humans Grasp-Related Areas Depend on Hand Used and Handedness

**DOI:** 10.1371/journal.pone.0003388

**Published:** 2008-10-10

**Authors:** Chiara Begliomini, Cristian Nelini, Andrea Caria, Wolfgang Grodd, Umberto Castiello

**Affiliations:** 1 Department of General Psychology, University of Padua, Padua, Italy; 2 Section experimental MRI of the CNS, Department of Neuroradiology, Tuebingen University Hospital, Tuebingen, Germany; Lund University, Sweden

## Abstract

**Background:**

In non-human primates grasp-related sensorimotor transformations are accomplished in a circuit involving the anterior intraparietal sulcus (area AIP) and both the ventral and the dorsal sectors of the premotor cortex (vPMC and dPMC, respectively). Although a human homologue of such a circuit has been identified whether activity within this circuit varies depending on handedness has yet to be investigated.

**Methodology/Principal Findings:**

We used functional magnetic resonance imaging (fMRI) to explicitly test how handedness modulates activity within human grasping-related brain areas. Right- and left-handers subjects were requested to reach towards and grasp an object with either the right or the left hand using a precision grip while scanned. A kinematic study was conducted with similar procedures as a behavioral counterpart for the fMRI experiment. Results from a factorial design revealed significant activity within the right dPMC, the right cerebellum and AIP bilaterally. The pattern of activity within these areas mirrored the results found for the behavioral study.

**Conclusion/Significance:**

Data are discussed in terms of an handedness-independent role for the right dPMC in monitoring hand shaping, the need for bilateral AIP activity for the performance of precision grip movements which varies depending on handedness and the involvement of the cerebellum in terms of its connections with AIP. These results provide the first compelling evidence of specific grasping related neural activity depending on handedness.

## Introduction

The highly developed ability of the hand to grasp and manipulate objects under precise visual control is one of the key features of the human motor system. In recent years, there have been significant advances in our understanding of the neural mechanisms underlying the motor commands that allow the hand to be shaped for efficient grasp of the object. An important step forward comes from studies in which single neurons were recorded during reach-to-grasp actions [1]. These studies showed that in macaques grasp-related sensorimotor transformations are accomplished in a circuit constituted by the anterior-most region within the lateral bank of the intraparietal sulcus (area AIP), the ventral premotor area F5 and the dorsal premotor area F2 [2]. It is postulated that AIP may furnish area F5 with visual signals of objects to aid in the selection of grasp configurations that are appropriate for their intrinsic attributes (e.g., size). Then F5 provides grasp-related information to the dorsal premotor area F2. Area F2 contains neurons with similar properties as those found in F5 in terms of types of grasp discrimination during movement execution and it has the role to monitor hand configuration while the to-be-grasped object is approached [2]–[4].

Many neuroimaging studies have explored in humans the existence of a cortical grasping circuit similar to that described in monkeys revealing activation within the putative homolog of macaque areas AIP, F5 and F2 for reviews see [5], [6]. All these neuroimaging studies, however, have so far been focused on reach-to-grasp movements of the right hand in right-handers participants. An issue, which has yet to be addressed is concerned with how the neural circuit underlying grasping modulates with respect to handedness, a basic feature of the human motor behavior. To date, the only available evidence comes from an unpublished report in which seven right-handed subjects performed grasping to visual targets with either the right or the left hand while scanned [7]. The main result was that grasping with either hand led to bilateral AIP activation, though both the extent and the magnitude of activation were much larger in the hemisphere contralateral to the hand used.

Although this preliminary study provides some hints regarding the lateralization of grasping related activity in right-handed humans, there is a call for a full investigation which considers possible differences across hands in both right- and left handed subjects. Such comparison becomes particularly relevant when considering recent behavioural evidence comparing the performance of right- and left-handers in a precision grip task. The results show that whereas right-handers exhibited a strong preference to use their dominant right hand to grasp the objects, left-handers did not show this preference and instead used their right hand 50% of the time [8], [9]. These results suggest that the hand used for precision grasping does not follow the usual pattern of asymmetries in hand use that defines left and right handedness.

To our knowledge, there are no published studies that have investigated whether brain activation for grasping tasks is always contralateral to the hand, modulated by handedness and the used hand., or always located in the same hemisphere regardless of handedness. Therefore, here we studied the kinematics and fMRI activation patterns of right- and left-handers humans during the performance of a precision grip movement (PG) executed with either the right or the left hand (RH, LH, respectively).

## Results

### Functional MRI

Blood Oxygenated Level Dependent (BOLD) signal was measured during the execution of PG movements performed with either the right or the left hand, by right-or left-handed participants. An analysis of variance (ANOVA) with handedness (right-handers, left-handers) as a between-subjects factor and performing hand (RH, LH) as a within-subjects factor was performed. Data were analyzed by applying a voxelwise analysis within a brain mask including regions selectively involved in visually guided precision grasping in humans [5], [6]. Specifically, these areas were the primary motor cortex (M1), the dorsal and ventral regions of the premotor cortex (vPMC and dPMC, respectively), the anterior part of the intraparietal sulcus (AIP) and the cerebellum [5], [6].

#### Main effect of handedness

When contrasting activity related to handedness independently from the used hand (right-handers/RH+right-handers/LH)>(left-handers/RH+left-handers/LH) significant differential activity was found within the primary motor cortex bilaterally (see Table 1). The opposite contrast (left-handers/RH+left-handers/LH)>(right-handers/RH+right-handers/LH) did not bring to any significant effect.

**Table 1:** Brain regions showing significant effects for the ANOVA handedness by performing hand.

Cluster level		Voxel level			MNI			SIDE	AREA	%
P(corr)	K	p (FWE)	T	Z	x	y	z			
**Main effect of handedness**										
***(Right-handers/RH+Right-handers/LH)>(Left-handers/RH+Left-handers/LH)***										
0.007	18	0.038	4.79	4.36	−43	−17	51	L	PreCG (4a)	70
0.049	84	0.045	4.66	4.26	40	−23	51	R	PreCG (4a)	50
**Main effect of performing hand**										
***(Right-handers/RH+Left-handers/RH)>(Right-handers/LH+Left-handers/LH)***										
0.000	395	0.000	9.66	7.36	−36	−20	57	L	PreCG (4a)	70
		0.000	8.81	6.93	−43	−20	54	L	PreCG (4p)	50
***(Right-handers/LH+Left-handers/LH)>(Right-handers/RH+Left-handers/RH)***										
0.000	212	0.000	8.16	6.58	40	−23	57	R	PreCG (4a)	70
		0.000	7.70	6.32	36	−26	69	R	dPMC (6)	90
**Interaction handedness by performing hand**										
***(Right-handers/RH−Right-handers/LH)−(Left-handers/RH−Left-handers/LH)***										
0.000	24	0.001	5.74	5.06	33	−17	69	R	dPMC (6)	100
0.018	61	0.021	4.98	4.51	−46	−44	48	L	AIP (40)	60
0.003	34	0.030	4.88	4.43	42	−45	48	R	AIP (40)	60
0.000	10	0.030	4.88	4.43	20	−53	−27	R	Cerebellum (6^th^)	

*Notes.* Only activations detected with the Small Volume Correction applied to the random effect analysis were considered. For each local maxima number of activated voxels (k), T and Z values, MNI coordinates and statistical significance (p<0.05 FWE corrected) for t-tests comparisons are reported (for both cluster-and voxel-level). Anatomical specifications are based on cytoarchitectonic probabilistic maps and the corresponding probability (%) values are reported. (FWE corrected, p<0.05 within the mask). (L = left; R = right). AIP = anterior intraparietal sulcus; dPMC = dorsal premotor cortex; PreCG = Precentral gyrus.

#### Main effect of performing hand

The contrast testing for differences between RH and LH independently from handedness (right-handers/RH+left-handers/RH)>(right-handers/LH+left-handers/LH) revealed significant activity within both the anterior and posterior sectors of the left primary motor cortex (see Table 1). The opposite contrast (right-handers/LH+left-handers/LH)>(Right-handers/RH+Left-handers/RH) revealed significant differential activity within the right primary motor and dorsal premotor cortices (see Table 1).

#### Interaction handedness by performing hand

**Figure 1:**
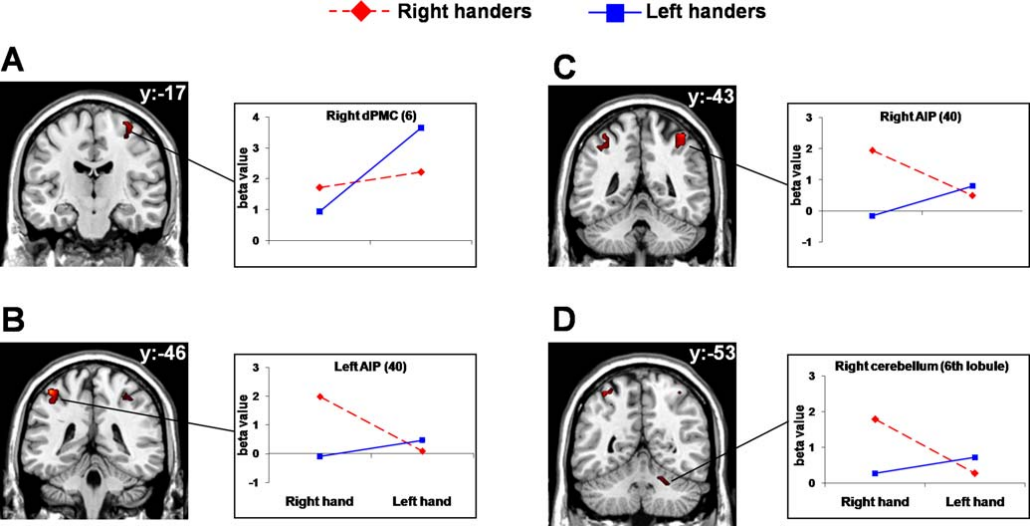
Functional MRI results. Group statistical map resulting from the interaction between handedness and performing hand. The contrast (Right-handers/RH−Right-handers/LH)−(Left-handers/RH−Left-handers/LH) revealed significant effects on brain activity within the dPMC (A), the left (B) and the right (C) AIP, and the right cerebellum (D), corresponding to the 6^th^ lobule. Only activations detected with the Small Volume Correction applied to the random effects analysis are considered (FWE corrected, p<0.05 within the mask). Side panels report the beta values observed for the indicated area. Anatomical specifications are defined on the basis of cytoarchitectonic probabilistic maps (Eickoff et al, 2007). Activation maps are superimposed on the ch2 template provided with the software package MriCRO. Images are displayed in neurological convention. MNI coordinates for the significant areas are reported in Table 1.

The interaction between handedness and performing hand (right-handers/RH−right-handers/LH)−(left-handers/RH−left-handers/LH) revealed significant differential activity within the right hemisphere for the dPMC and the cerebellum (6^th^ lobule). Furthermore, significant activity was observed bilaterally within the AIP. For the dPMC, post-hoc contrasts revealed that activity within this area was greater for left- than right-handers when the left hand was used to perform the task (Fig. 1a and Table 1). Such difference was not evident when the right hand was used. Further, whereas left-handers showed a significant increase in activation for the left in respect to the right hand, right-handers showed a similar level of activity for the two hands. When exploring the significant variations of brain activity detected within the AIP bilaterally (Fig. 1b–c and Table 1), AIP was significantly more activated in right-handers in respect to left-handers when the performing hand was the right. In addition, in right-handers the use of the right dominant hand led to stronger activity in respect to the left hand within AIP in both hemispheres. No differences were found concerning left-handers and between right- and left-handers when using the left hand. A similar pattern of activation was found for the 6^th^ lobule of the right cerebellar hemisphere (Fig. 1d and Table 1). The *t*-contrast (right-handers/LH−right-handers/RH)−(left-handers/RH−left-handers/LH) did not bring to any significant effect.

### Behavioural Experiment

For each subject the average value for each kinematical measure across trials was entered into an ANOVA similar to that performed for the fMRI data.The analyses were performed on the dependent variables considered as ‘classic’ markers of reach-to-grasp kinematics: (i) movement duration; (ii) the amplitude of maximum arm peak velocity; (iii) the time of maximum arm peak velocity; (iv) the time of maximum grip aperture (i.e., the maximum distance between the thumb and index finger); (v) the time from maximum grip aperture to the end of the movement (closing time) and (vi) the amplitude of maximum peak grip aperture. Given the rather mixed results obtained by the paucity of studies comparing reach-to-grasp kinematics of the right and left hand in right-handers and the lack of previous evidence concerned with similar behaviors in left-handers, it is rather difficult to make specific prediction on how the considered variables will behave. If the right and the left hand will follow the same kinematic structure, as some studies on right-handers have demonstrated [10]–[14], then we may not expect differences related to handedness for the considered kinematic variables. Alternatively, if the results that right-handers reach out to grasp stimuli with an unusual hand posture when using the left rather than the right hand is confirmed [12], [13] then we expect a reverse pattern in left-handers. For instance we could expect that right-handers would manifest a less dexterous performance with the left hand and when the less dexterous hand is used a wider safety margin would be put in place. Such effect should be evident in a larger and anticipated maximum grip aperture, a faster initial hand opening and increased time from the time of maximum grip aperture to the end of grasp and an overall increase in movement duration. Further, if the suggestion that left-handers are more likely to use the right than the left hand to perform precision grasp movements [8], [9], therefore violating handedness, then we may expect the left hand to behave less dexterously than the right hand for this subjects' group.

**Figure 2:**
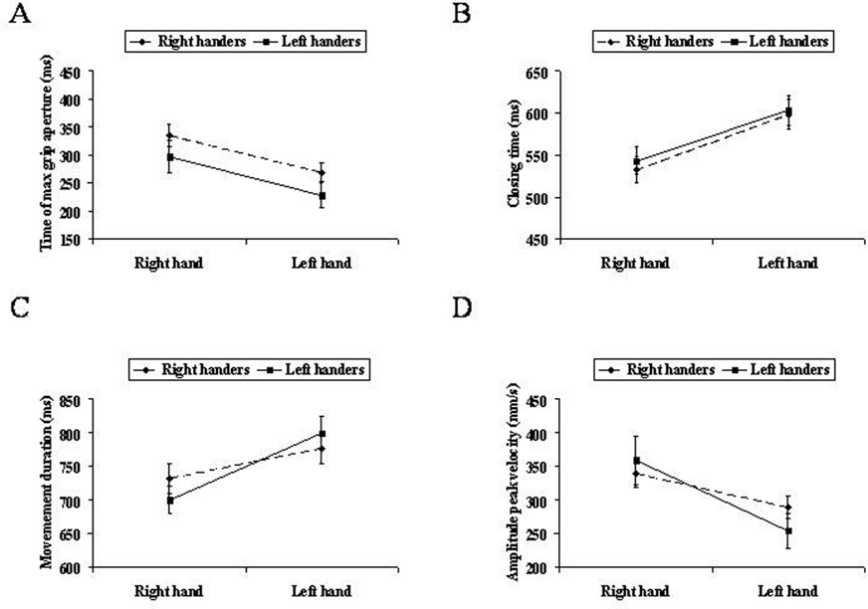
Kinematics results. Graphical representation for the main factor ‘hand’ for the time of maximum grip aperture (A) and closing time (B). Graphical representation for the interaction handedness by hand for movement duration (C) and the amplitude of peak velocity (D).

The main factor ‘performing hand’ was significant for the time at which the hand reached its maximum aperture [F(1,9) = 39.41, p<0.0001; η^2^_p_ = 0.87] and for closing time [F(1,9) = 32.05; p<0.0001; η^2^_p_ = 0.81], the time from the maximum grip aperture to object contact. For both right- and left-handers the time to maximum grip aperture was anticipated (Fig. 2a) and closing time was longer (Fig. 2b) when the task was performed with the left than with the right hand. The interaction between handedness and performing hand was significant for movement duration [F(1,9) = 16.13, p<0.0001; η^2^_p_ = 0.77] and the amplitude of peak velocity [F(1,9) = 16.22; p<0.001; η^2^_p_ = 0.67]. Post-hoc contrasts revealed that when left-handers used the left hand movement duration was longer (Fig. 2c) and the amplitude of peak velocity was lower (Fig. 2d) than when the right hand was used (p_s_<0.05). For right-handers movement duration (Fig. 2c) and the amplitude of peak velocity (Fig. 2d) were similar for both hands (p_s_>0.05). Though there was a strong tendency indicating that for this group movement duration was shorter and the amplitude of peak velocity faster for movements performed with the right hand (p = 0.057 and p = 0.06, respectively; see Fig. 2c–d).

## Discussion

We set out to compare neural activity and kinematics underlying precise grasping performed with either the right and the left hand by both right- and left-handers. Results indicate that overall both right- and left-handers accomplished the task equally well with either the right and the left hand. However, as outlined below, depending on the relationship between handedness and the hand used distinguishable neural and kinematic patterns were revealed.

### Activity Related to the Right Dorsal Premotor Cortex

On the basis of neurophysiological and neuroimaging studies the role of the dPMC for distal forelimb movements is becoming increasingly established [3], [15], [16]. In this respect, monkeys and human studies agree that the dPMC has the role of keeping in memory the motor representation of the to-be-grasped object and combine it with visual information as to continuously update the configuration and orientation of the hand as it approaches the object to be grasped [3], [16].

Here we show that it was only the dPMC within the right hemisphere which was significantly activated. A result which is in accordance with previous evidence suggesting a specific right hemisphere contribution to grip formation [17], [18]. Specifically, the pattern of activation shows that, whereas activity within the right dPMC was similar for both right- and left-handers when performing the task with the right hand, it differed between the two groups when the left hand was used. This was evident when looking at the significant increase in activation when left-handers used the dominant left rather than the right hand. A possible explanation for these effects might be found in recent neurophysiological and neuroimaging findings demonstrating that the dPMC is involved in the control of distal actions [2], [3], [4], [15], [16]. In first instance, one study provides compelling evidence that in the distal forelimb representation of area F2 there are neurons that are selective for the type of prehension required for grasping the object [3]. These results indicate an important role of the dPMC in the control of goal-related hand movements. Specifically, the proposal here is that the dPMC involvement during goal-directed actions might be highly correlated with the accuracy requirement of the ongoing movement [19]. In humans the contribution of the dPMC to hand movements, the time course of its involvement and its hemispheric dominance are essentially unknown. A recent neuroimaging investigation, however, suggests that in humans as in monkeys this area is involved in the control of grasping [16]. In this study the congruency between the adopted grasp and the grasp called by an object was investigated. An increase of activity within the dPMC for incongruent grasps was reported. In order to resolve the mismatch between type of grasp and object size which, occurred for the incongruent conditions, this area showed an increase of activation which was interpreted as the need for a more effective control. Assuming that, as previously demonstrated, left-handers show a significant tendency to use the right hand when performing precision grip movements [8], [9] such increase may signify that the dPMC was differentially activated because the dominant left-hand was less skilled as to perform the task and more control was needed.

The pattern of dPMC activity for the interaction between handedness and hand mirrors the pattern obtained for the same interaction for movement duration and the amplitude of maximum peak velocity. Remember that movement duration was longer and the amplitude of peak velocity was lower when left-handers used the left than the right hand. However, for right-handers no significant differences across hands were observed (though a slightly similar trend was noticed). Such ‘slowness’ may suggests that the dPMC is differentially activated when both right- and left-handers use the left rather than the right hand. Support for this contention comes when considering kinematics results in terms of the main effect of ‘hand’. We found a significant anticipation of maximum grip aperture and an increase in closing time for the left than for the right hand in both subjects' groups. These are two kinematic parameters indicative of the timing taken by the hand to establish adequate finger positioning before object lifting. Altogether these findings are in line with previous evidence suggesting that the dPMC may control the grasping phase before the lifting phase [15]. Because the lifting phase can only be initiated when finger positioning is completed and/or when the grip force has reached an adequate level, the present results concerned with the less dexterous hand are compatible with such a function of the dPMC.

### Bilateral Activity Within the Anterior Intraparietal Sulcus

In both humans and monkeys AIP is an important component of the parietal-premotor circuit known to be involved in the transformation of an object's intrinsic properties into specific grips [1], [6]. In our study, we confirm the pattern of AIP bilateral activity previously found in right-handers using either the right or the left hand [7], [20]–[24]. Although this result could be due to a bidirectional crosstalk between the two homologous areas or more simply to the evidence that we can grasp objects with two hands [25], recent evidence suggests that bilateral AIP activity is a pre-requisite for hand shaping formation [24]. By using transcranial magnetic stimulation (TMS) it has been revealed that whereas unilateral AIP virtual lesion failed to impair hand shaping, such process was impaired when TMS was applied bilaterally to AIP [24]. This suggests that both AIPs might be necessary regardless of the hand in use [24]. Further, indirect evidence, that a bilateral AIP virtual lesion might be needed to disrupt grasping comes from two studies revealing that a unilateral AIP lesion failed to alter the hand conformation except when the object size and orientation were unexpectedly changed [26], [27]. There is also evidence, however, that unilateral TMS over either the right or the left AIP disrupts grasping movements for the contralateral hand [28], [29]. A result which is in contrast with the findings reported above suggesting that a bilateral lesion of AIP might be necessary to determine a deficit in hand shaping [24]. Yet, a close inspection of the experimental protocols used in these studies suggest that such discrepancies can be accounted by the different timing at which the TMS pulses were applied. Whereas in the studies suggesting the need for a bilateral AIP virtual lesion to elicit grasping disruption the TMS pulses were applied during action execution [24], those studies revealing grasping disruption following unilateral TMS application delivered the pulse at the time the movement was planned [28], [29]. Because the fMRI results reported here are concerned with grasping execution, we are inclined to suggest that our findings provide further fuel to the proposal that a bilateral AIP involvement might be necessary for the execution of precision grip movements and might be a distinctive feature of the anterior sector of human posterior parietal cortex [5], [20]–[23], [30].

Noticeably in the present study the pattern of activity found within this area is similar in both hemispheres, but differs depending on hand and handedness. In particular, an increase of bilateral AIP activity was evident for right-handers with respect to left-handers when using the right-hand. For the two subjects' groups a similar level of activity was found for the left hand. These findings may suggest the superiority of the right hand in the performance of manipulations requiring dexterous finger movements, high precision in interjoint coordination and trajectory formation in right-handers [31], [32]. Therefore the accuracy demanded by the task used here and the consequent need for the determination of precise contact points may allow to reveal such right hand superiority in right-handers. In this view it would be tempting to suggest that such ‘superiority’ requires more sophisticated visuomotor transformation processes which translate in an increased level of activity in AIP when right-handers used the right hand for accurate tasks. Evidence that AIP activity might be modulated by the level of task complexity comes from both inactivation study in macaques [33] and neuroimaging studies [16], [34]. In first instance, transient inactivation of AIP, by injecting a GABA-receptor agonist (muscimol) produced grasping errors only for difficult tasks that required a precision grip to grasp a small cube or a small sphere (as in the present experiment). In second instance right-handed subjects show a significant increase in grasp related AIP activity when performing a precise than when performing whole hand grasp [16], [34].

Conversely, for left-handers a similar level of AIP activity for either hand was found. Therefore with a certain degree of caution we suggest that for left-handers the level of visuomotor trasformation occurring in AIP might be indistinguishable for the two hands. This observation, is in line with the anatomical observation of differences in interhemispheric connections in relation to handedness [35]. And it might also suggest differences in the functional organization of AIP in right- and left-handed people as previously demonstrated for motor and premotor areas [36].

### Right Cerebellar Activity

Lesion, imaging, and electrophysiological evidence suggest a cerebellar involvement during prehension. Cerebellar patients [37]–[42] exhibit a spectrum of kinematic impairments in the learning, planning, and execution of prehensile movements which are consistent with the proposal that the cerebellum plays a major role in the control and coordination of reach-to-grasp movements. Further, neurophysiological work has identified various cerebellar structures implicated in the kinematics of reach-to-grasp movements [43]–[47]. Similarly, functional imaging reach-to-grasp studies reported cerebellar activations [34], [48]–[50].

Of interest is that in the present study the pattern of activity found for the right cerebellum mirrors that found for AIP. This result may be explained in light of recent developments for the investigation of the anatomical connections between key grasping areas such as AIP and the cerebellum. Specifically, using retrograde transneuronal transport of viruses cerebellum inputs to AIP have been revealed [51]–[54]. Therefore the similarity of the pattern of cerebellar activity with the pattern of activity found in AIP (and their possible connections) expands the potential sphere of influence of the cerebellum. This adds an additional layer of complexity to this picture by possibly demonstrating that AIP receives input from the cerebellum which is necessary for the adaptive adjustment of motor output and sensorimotor mechanisms which could have great utility for adjusting hand shape during object manipulation. The result that it seems to be chiefly the right cerebellum to be involved confirms the specificity of the right hand for the performance of precise grasping actions.

### Kinematic Observations

Previous studies on grasping comparing the performance for the right and the left hand are largely confined to the right-handers population [13], [55]. In keeping with these previous findings the present results confirm that in right-handers both the right and the left hand largely share a common level of kinematic parameterization [13], [55]. Further, they indicate that in right-handers movements performed with the right hand were generally faster and the left hand grasped with a wider safety margin. In this respect, the time of maximum grip aperture was anticipated and closing time was increased for the left than for the right hand. This suggests that when the left hand was used more time and on-line control was needed as to possibly compensate for a higher end-point variability. This is in accordance with previously published data in right-handers suggesting that manipulations requiring dexterous finger movements, such as precision grip, are mastered more efficiently by the right hand [13], [31], [55].

Although the results for left-handers largely mirror those obtained for right-handers, an important observation is that in left-handers the use of the left hand dictated the put in place of compensatory strategies (e.g., anticipation of the time of maximum grip aperture) which were similar to those adopted by right-handers when using the same hand. We suspect that this occurs because grasping a small object with a precision grip with the left hand might be for the left-handers group a quite unnatural act. This idea is supported by behavioral observations on both human [8], [9] and non-human primates [56]. In first instance left-handers are much more likely than right-handers to use the “non-dominant” hand to pick up objects [8], [9]. In second instance, a series of elegant studies on hand preferences in chimpanzees has reported that chimpanzees who used a precision grip to grasp small pieces of food were more likely to use their right hand [56]. Therefore the preferential use of the right hand by left-handers in a precision grip task may reflect a property of the brain that is ancient and hard-wired as the studies on hand preferences in chimpanzees, our closest phylogenetic relatives, may demonstrate.

### Conclusions

The present results shed new light on the functional mechanisms presiding over the control of visually guided hand grasping actions in both right- and left-handers. Specifically, the strength and the novelty of our findings come chiefly from contrasting both hands in these populations. This enabled us to define the functional properties of key areas involved in the control of grasping depending on handedness. Crucially, they extend the current human neuroimaging literature in three important and interconnected ways. First, they highlight the role played by the right dPMC in monitoring the configuration of fingers when precise prehensile movements are performed by either the right and the left hand. This role becomes particularly evident when the hand less-skilled to perform such action is utilized. Second, they provide behavioural and neuroimaging evidence that both right- and left-handers prefer the right hand when precise grasp has to be performed. Finally they offer some indirect evidence in humans of the connections between the cerebellum and AIP, an area which is fundamental for the visuomotor transformations underlying grasping.

## Materials and Methods

### Functional MRI

#### Subjects

Nineteen right-handed (12 women and 7 men; age range: 19–30 years; mean age: 24,7 years) and fifteen left-handed (10 women and 5 men; age range: 21–35 years; mean age: 26,1 years) participated in the experiment. They all had normal or corrected-to-normal vision, and they had no neurologic or psychiatric history, or any motor pathology. Handedness (right-handedness, left-handedness) was assessed by using a test of manual dominance [57]. On the basis of the scores obtained with this test all right handed participants were classified as strongly right handed (36/36) and all left handed participants were classified as strongly left handed (36/36). Before entering the scanner room all gave informed written consent according to the Tuebingen University Hospital Ethical guidelines and with the declaration of Helsinki.

#### Stimulus

The stimulus consisted of a spherical plastic objects of 3 cm diameter presented at a constant distance of 30 cm. We used a regular geometric shape rather than functional objects (i) for comparability with macaque neurophysiology studies [33], [58] and (ii) to examine grasping in a general manner, rather than introducing further aspects like tool use involving a particular network in the left-hemisphere [59]. Stimulus dimension was chosen in order to elicit a PG type of prehension which considers the opposition of the pulpar surface of the index finger with the thumb. We decided to confine our analysis to PG movements because it is more demanding than other type of prehensile movements in terms of accuracy and neural processing, as demonstrated in previous kinematic and neuroimaging studies [5], [6].

#### Experimental setup

Stimulus was presented by means of a metal free structure, allowing for presentation of 3D stimuli in the scanner bore [16], [34]. The device was attached to the sides of the sliding bed and was fitting the diameter of the bore. Two sliding bars allowed for the regulation of stimulus position, in order to present it at waist-deep, easily and comfortably reachable from participants' hand while lying down in the scanner bore, without the need for upper arm or shoulder movement. To further minimize the risk of head movement, potentially induced by arm movements, upper arms were fixed with an elastic band. In order to maintain constant the hand starting position across subjects and trials subjects wore a metal-free belt upon which a pad was attached. At the start of each trial the performing hand (right or left) was maintained in a relaxed position laying with the palm upon the pad whereas the other upper arm/hand ensemble was stripped to the scanner bore. The head was tilted at an angle (∼30 deg) and supported with a foam wedge, that permitted direct viewing of the stimuli below the coil without mirrors. Such direct viewing avoids introducing additional transformations required by mirror-viewing [23], [60], [61]. In addition, participants were allowed free viewing between trials, but they were explicitly instructed to look at the object during action execution.

#### Task procedures

The experiment was conducted within an illuminated room. Participants were requested to perform two types of action: grasping the object with a precision grip through the opposition of thumb and index finger (G), or to simply reach it (R), touching it with the back of the hand by using a closed fist posture. The reaching action served two purposes: First, it served as control condition at first level analysis (see ‘First-level data analysis’ section). Second, it prevented the possible occurrence of fMR adaptation concerning action execution. Participants were requested to perform the movements with either the right (RH) or the left (LH) hand. Participants were instructed to unfold the action at a natural speed and were informed about the action to perform through a sound delivered by pneumatic MR-compatible headphones: (i) G - low tone (duration: 200 ms; frequency: 1,7 kHz); (ii) R - high tone (duration: 200 ms; frequency: 210 Hz.). Although the object was always visible, participants were explicitly requested to start the movements at the time the sound was presented. Therefore, the sound served both as a ‘go’ signal and as to indicate the type of action (R or G) to be performed. From the control cabin beside the scanner room it was possible to monitor the person inside the scanner through a glass. Therefore the it was possible to control whether the subjects responded to the sounds and whether they were performing the action corresponding to the presented tone.

#### Experimental design

The experiment was conducted by using a mixed event-related design. Handedness (right-handers, left-handers) was the between-subjects factor. Type of action (G, R) and performing hand (RH, LH) were the within-subjects factors. The type of action was alternated within runs by following a pseudo-randomized sequence. Trials of the same type (R or G) were grouped in sequences varying from four to eight elements. This was done in order to minimize brain activity due to frequent task changes [61]. The performing hand (LH, RH) was maintained constant within runs, and alternated between runs. Further, Inter Stimulus Interval (ISI) varied from 3 to 8 seconds following a ‘long exponential’ probability distribution [62] and was randomized across trials. A total of 70 trials per each condition was administered. The whole experimental session consisted of 280 trials, divided into 4 runs of 70 trials each. Runs were kept very short in order to avoid participant's fatigue.

#### Imaging parameters

Images were acquired with a whole-body 3 T scanner (Siemens Magnetom Trio, TIM system) equipped with a standard Siemens 12 channels coil. Functional images were acquired with a gradient-echo, echo-planar (EPI) T2*-weighted sequence in order to measure blood oxygenation level-dependent (BOLD) contrast throughout the whole brain (47 contiguous axial slices acquired with descending interleaved sequence, 64×64 voxels, 3.3×3.3×3 mm resolution, FOV = 210×210 mm^2^, flip angle = 90°, TE = 30 ms, bandwidth:1954 Hz/Px). Volumes were acquired continuously with a repetition time (TR) of 3 s; 77 volumes were collected in each single scanning run (3:51 minutes). High-resolution T1-weighted images were acquired for each subject (3D MP-RAGE, 176 axial slices, data matrix 256×256, 1 mm isotropic voxels, TR = 1859 ms, TE = 3.14 ms, flip angle = 22°).

#### First-level data analysis

Data analysis was performed using the software package SPM5 (Wellcome Department of Imaging Neuroscience, University College of London, UK - http://www.fil.ion.ucl.ac.uk/spm). The first four scans for each session were excluded from data analysis because of the non-equilibrium state of magnetization. For each participant, images underwent motion correction, and each volume was realigned to the first volume in the series. The anatomical scan was then co-registered to the mean of all functional images, previously corrected for intensity inhomogeneities through the bias correction algorithm implemented in SPM5. EPI images were then normalized (resampling: native voxel size) adopting the MNI152 template, supplied by the Montreal Neurological Institute (http://www.mni.mcgill.ca/) and distributed with the software SPM. Finally images were smoothed using a 6.6×6.6×6 mm FWHM 3D Gaussian kernel (twice the native voxel size). High-pass filtering (128 sec) was also applied to remove low-frequency drifts in signal. After motion correction four participants (three right-handers and one left-hander) had to excluded from further analysis because of large head motion (exceeding voxel size, 3.3 mm). At the first level, for each single subject, types of action (G, R) performed with either the right or the left hand (RH, LH) were modelled as separate events type. The duration was assumed of about 2 seconds on the basis of behavioural observations before the experimental session. This was done in order to get participants acquainted with the experimental setup. Regressors were defined on the timing of presentation of each experimental condition. These functions were convolved with a canonical, synthetic HRF (haemodynamic response function) and its first-order temporal derivative to produce individual models [63]. The temporal derivative was considered since it allows for a temporal shift of the peak of the BOLD response. Errors (incorrect actions) were modelled as a further condition of no interest (maximum error rate: R = 0.6%; G = 1,2%). For each subject, all regressors were incorporated into General Linear Models [GLM – 64] and motion correction parameters, created during the realignment stage, were included in the analysis as a covariate of no interest. This was done in order to model residual effects due to head motion. Individual models were separately estimated and contrasts were defined in order to pick out the main effects of each experimental condition. Then, for each participant the reaching-related activation was subtracted from the correspondent grasping-related activation (G_RH>R_RH; G_LH>R_LH). This procedure has been adopted in several previous neuroimaging studies on visuomotor control of grasping in humans [16], [34], [61], [65] as to isolate brain activation solely related to the hand shaping process involving the palm and the fingers while approaching and grasping the object. The subtraction was applied for each participant.

#### Second-level data analysis

HRF contrasts resulting from the subtraction (e.g., G_RH>R_RH) performed at the first level analysis were then entered into a second level random-effect analysis (2×2 ANOVA) in which performing hand (LH or RH) was manipulated as within-subjects variable (corrected for sphericity and equal variance assumed), and handedness (Right-handers, Left-handers) served as a between-subjects variable. The resulting SPM*{t}* maps reflected areas in which variance related to the experimental manipulation was captured by the HRF adopted in the GLM.

As outlined at the start of the ‘Results’section, we focussed our investigation on the specific contribution of areas involved in the execution of grasping movements. To date only a preliminary imaging study has compared reach-to-grasp movements performed with the right and the left hand in right-handers [7] showing bilateral AIP activity. Therefore we may expect a similar result in the present study for right-handers which may extend to left-handers. With respect to premotor cortices we cannot make any firm prediction. However, considering the evidence of a specific role for the dPMC in monitoring the configuration and orientation of the hand as it approached the to-be-grasped object [15], [16] it may be expected that this area may show a differential activation in both right- and left-handers for the control of the non-dominant hand (i.e., left and right hand, respectively). A caveat concerned with this prediction is that if, as previously demonstrated, left-handers prefer to use the non-dominant hand to perform precision grasp tasks, then this area should be more involved when the left hand is used.

We considered an anatomy-based mask, involving three-dimensional cytoarchitectonic probabilistic maps of premotor and motor cortices [66], [67] together with the anterior bank of the intraparietal sulcus [68] for both the left and the right hemispheres. All these maps are implemented with the Anatomy Toolbox [69], [70]. Moreover, the three-dimensional anatomic map for both cerebellar hemispheres, obtained through the anatomical parcellation of the MNI spatially normalized single-subject high-resolution T1 template [71], was included in the mask. A global mask involving all these areas was created with the “imCalc” function implemented in SPM5. Then the mask was adopted as a searching area [Small Volume Correction, SVC – 72]; only activations surviving the threshold of FWE .05 within the mask and associated with a probability value equal or greater than 60% within the respective cytoarchitectonic map were considered [70]. Anatomical labeling of fMRI results will refer to cytoarchitectonic maps.

### Behavioural Experiment

#### Subjects

Twenty participants (7 men, 13 women; mean age 25.5±3 years) volunteered to participate. Ten participants showed right-handed dominance (4 men, 6 women; mean age 25.8±2.86 years) whereas ten showed left-handed dominance (3 men, 7 women; mean age 25.2±3.26 years). Handedness was determined using a test for manual dominance [57]. None reported visual or psychomotor dysfunction. All subjects were naïve as to the experimental design or purpose and gave their informed consent to participate in the study. The experimental procedures were approved by the Institutional Review Board at the University of Padua and were in accordance with the declaration of Helsinki.

#### Apparatus and procedures

The stimulus, the apparatus and the procedures were similar in all respects to those described for the fMRI experiment except that here a purely reaching condition was not considered. Infrared reflective markers (0.25 cm diameter) were taped to the following points on the subjects' upper limbs: (1) wrist – dorsodistal aspect of the radial styloid process; (2) thumb – ulnar side of the nail; and (3) index finger – radial side of the nail. Markers were fastened using double-sided tape. Movements were recorded using an ELITE motion analysis system (Bioengineering Technology & Systems [B|T|S]). Four infrared cameras (sampling rate 100 Hz) placed 120 cm away from each of the four corners of the table captured the movement of markers in 3D space. Coordinates of the markers were reconstructed with an accuracy of 0.2 mm over the field of view. The standard deviation of the reconstruction error was 0.2 mm for the vertical (Y) axis and 0.3 mm for the two horizontal (X and Z) axes. The experimenter was given on-line computer screen feedback of the three-dimensional position of each marker – if one marker was missing during task performance the trial was manually discarded. Experimentation continued until the required number of successful trials was collected (N = 10) for each experimental condition (right handers/right hand; right-handers/left hand; left handers/right hand; left-handers/left hand).

#### Data processing

The ELIGRASP software package (B|T|S|) was used to analyze the data and provide a 3-D reconstruction of the marker positions as a function of time. The data were then filtered using a finite impulse response linear filter (transition band = 1 Hz, sharpening variable = 2, cutoff frequency = 10 Hz). Following this operation, the tangential speed data for the wrist marker were used to determine the onset of the movement using a standard algorithm (threshold for movement onset was ∼5 cm/s). Movement onset was taken as the earliest time at which movement of the wrist exceeded the set threshold. Movement offset was taken at the latest time at which the movement of the thumb and index finger occurred. For each subject the average value for the considered dependent measure (please refer to the ‘Results’ section for the behavioural experiment) across trials was entered into an ANOVA with handedness (right-handers, left-handers) as a between-subjects factor and performing hand (RH, LH) as a within-subjects factor. Bonferroni corrections were applied to the contrasts of interest (throughout the text significant values are indicated). Preliminary analyses were conducted to check for normality, univariate and multivariate outliers, with no serious violations noted.
